# In vivo measurement of intradiscal pressure changes related to thrust and non-thrust spinal manipulation in an animal model: a pilot study

**DOI:** 10.1186/s12998-022-00445-1

**Published:** 2022-09-06

**Authors:** William R. Reed, Michael A. K. Liebschner, Carla R. Lima, Harshvardhan Singh, Christopher P. Hurt, Daniel F. Martins, James M. Cox, Maruti R. Gudavalli

**Affiliations:** 1 Rehabilitation Science Program, Palmer College of Chiropractic, Davenport, IA USA; 2 Present Address: Department of Physical Therapy, University of Alabama at Birmingham, Birmingham, AL USA; 3 Department of Medicine, Baylor College of Medicine, Houston, TX USA; 4 Rehabilitation Science Program, University of Alabama at Birmingham, Birmingham, AL USA; 5 Postgraduate Program in Health Sciences, University of Southern Santa Catarina, Palhoça, Santa Catarina Brazil; 6 Clinician Private Practice, Fort Wayne, IN USA; 7 College of Chiropractic Medicine, Keiser University, West Palm Beach, FL USA

**Keywords:** Intradiscal pressure, Intervertebral disc, Chiropractic, Spinal manipulation, Lumbar

## Abstract

**Background:**

The intervertebral disc is a known back pain generator and is frequently the focus of spinal manipulative therapy evaluation and treatment. The majority of our current knowledge regarding intradiscal pressure (IDP) changes related to spinal manual therapy involves cadaveric studies with their inherent limitations. Additional in vivo animal models are needed to investigate intervertebral disc physiological and molecular mechanisms related to spinal manipulation and spinal mobilization treatment for low back disorders.

**Methods:**

Miniature pressure catheters (Millar SPR-1000) were inserted into either the L4-L5 or L5-L6 intervertebral disc of 3 deeply anesthetized adult cats (Oct 2012-May 2013). Changes in IDP were recorded during delivery of instrument-assisted spinal manipulation (Activator V® and Pulstar®) and motorized spinal flexion with/without manual spinous process contact.

**Results:**

Motorized flexion of 30° without spinous contact decreased IDP of the L4-L5 disc by ~ 2.9 kPa, while physical contact of the L4 spinous process decreased IDP an additional ~ 1.4 kPa. Motorized flexion of 25° with L5 physical contact in a separate animal decreased IDP of the L5-L6 disc by ~ 1.0 kPa. Pulstar® impulses (setting 1–3) increased IDP of L4-L5 and L5-L6 intervertebral discs by ~ 2.5 to 3.0 kPa. Activator V® (setting 1–4) impulses increased L4-L5 IDP to a similar degree. Net changes in IDP amplitudes remained fairly consistent across settings on both devices regardless of device setting suggesting that viscoelastic properties of in vivo spinal tissues greatly dampen superficially applied manipulative forces prior to reaching deep back structures such as the intervertebral disc.

**Conclusions:**

This study marks the first time that feline in vivo changes in IDP have been reported using clinically available instrument-assisted spinal manipulation devices and/or spinal mobilization procedures. The results of this pilot study indicate that a feline model can be used to investigate IDP changes related to spinal manual therapy mechanisms as well as the diminution of these spinal manipulative forces due to viscoelastic properties of the surrounding spinal tissues. Additional investigation of IDP changes is warranted in this and/or other in vivo animal models to provide better insights into the physiological effects and mechanisms of spinal manual therapy at the intervertebral disc level.

## Background

Low back pain (LBP) is a complex, costly, and poorly managed global health problem with lifetime prevalence rates as high as 84% [1]. In a study involving 195 countries, LBP was identified as the leading cause of years lived with disability and productivity loss as measured in years [2]. Identifying the anatomical source of LBP is diagnostically challenging but the intervertebral disc is a known LBP generator [3–6] and frequently becomes a primary focus in manual therapy clincial evaluation and treatment [7, 8]. Most clinical guidelines recommend spinal manual therapies (high velocity low amplitude spinal manipulation and low velocity low amplitude oscillatory spinal mobilization) as conservative, non-surgical treatments for LBP with and without disc involvement [9–12]. Both clinical imaging and biomechanical studies clearly demonstrate bidirectional propogation of applied spinal forces and resultant vertebral movement throughout the spine emanating from the applied physical contact site during spinal manual therapy [13–16]. While spinal manual therapy forces have been shown to result in considerable loads being conveyed through the cadaveric disc, [17] to date most of our knowledge pertaining to the physiological impact of these forces on intervertebral disc tissue (i.e. intradiscal pressure, IDP) arises from human/animal cadaveric studies [18–25]. Much less is known regarding in vivo mechanical, physiological, and molecular changes related to spinal manual therapy in either healthy or pathophysiological intravertebral disc tissue [26]. Results from a in vivo human study in which L3/4 IDP was measured in a single healthy inidividual during a manually delivered spinal manipulation reported baseline IDP pressures in the prone and side-lying positions to be 110 kPa and 150 kPa respectively [27]. Peak intradiscal pressures of 662 kPa over 290 ms (without spinal joint cavitation) and 890 kPa over 125 ms (with joint cavitation) were recorded in this particular subject during spinal manipulative thrusts delivered in a side-lying position [27]. These peak pressures were noted to be similar in magnitude to healthy subjects while sitting upright (550–623 kPa) or sitting in a flexed position (830–1133 kPa) [28, 29]. Recognized limitations of this human in vivo study included a small sample size (n = 1), lack of any disc pathology, only a single manual therapy technique evaluated, the duration of spinal manipulation induced IDP changes were not determined, nor were physiological/biological mechanisms identified for any IDP changes related to spinal manipulation [27].

From cadaveric studies such as a recent study by Wang et al. [30] comparing cadaveric human lumbar disc pressure characteristics during simulated spinal manipulation and spinal mobilization, no differences in maximal IDP were reported between spinal manipulation (1050 ± 0.13 kPa) and spinal mobilization (1057 ± 0.11 kPa) using representative spinal loading parameters as determined from the literature. They found that the ascending speed of IDP during spinal manipulation was significantly faster than spinal mobilization and this distinctive biomechanical characteristic of spinal manipulation has long been theorized to uniquely influence physiological and subsequently therapeutic responses in the disc and surrounding spinal tissues [31]. Wang et al. also reported that the two types of simulated manual therapy increased peak IDP by 33 to 58% and maximum IDP differed between the rotating (ipsilateral) and contralateral side in the nucleus pulposus [30]. In general, these findings support work by Kawchuk and colleagues using a parallel robot and serial cadaveric dissection [17, 22, 25, 32]. These investigators recently reported that the peak resultant force experienced by the spinal segment corresponded to 12.1% of total applied spinal manipulation force of 300 N, and that the intervertebral disc experienced 99% of the peak and 96.5% of the mean forces reaching the spinal segment in a fresh porcine cadaveric model [25]. However, inherent limitations of in vitro and cadaveric spinal manipulative therapy studies include extensive trunk tissue disruption or removal, direct delivery of simulated manipulative forces to an isolated or potted spine, and loss of force dampening and functional response related to the viscoelastic and/or hydrostatic properties of surrounding tissues. Biomechanical studies of in vitro spinal segments indicated that spinal flexibility is significantly dependent on the axial loading. With in vivo studies the musculoskeletal system remains intact, however with in vitro studies, the intervertebral disc (IVD) tends to swell thereby generating an exacerbated flexibility beyond normal limits, which may alter the response of spinal segments to spinal manipulative therapy [33]. Taken together, these in vitro limitations prove to be significant barriers to determining mechanical, physiological, and molecular response mechanisms to spinal manual therapy in healthy and/or pathological discs. Development of new, minimally invasive [34] in vivo intervertebral disc models in small and large animals will contribute tremendously to overcoming many of the aforementioned cadaveric-related barriers in the study of physiological mechanisms associated with various spinal manual therapy procedures.

Among the few in vivo studies investigating spinal manual therapy dosage in disc degenerative models, Colloca et al. [35] examined neurophysiological responses (electromyographic activation and compound action potentials) following simulated spinal manipulation in a *disc degeneration* ovine model. Incisions (5 mm) were made in the posterolateral annulus fibrosus at L1-L2 under fluoroscopic guidance and with 20 weeks post-surgery allowing adequate time for these degenerative annular lesions to mature. Neurophysiological responses to spinal manipulative impulses (20 N, 40 N, 60 N, 80 N thrust magnitude and 10 ms, 100 ms, 200 ms thrust duration) were then recorded to mimic instrument-assisted (< 10 ms) and manually (100–200 ms) delivered spinal manipulation. Sheep in the degenerative lumbar disc group exhibited a 20–25% reduction in positive EMG responses and a 4.5–10.2% increase in compound action potential instantaneous frequency response as a result of spinal manipulation compared to control. While mechanical changes in the disc related to applied spinal manipulative impulses were not evaluated in this particular study, certain biomechanical quantifications such as vertebral movement were investigated in a subsequent study. In a follow-up study, Colloca et al. [36] reported that 100 ms manipulative thrusts resulted in significantly reduced vertebral displacements in sheep with lumbar disc degeneration and confirmed the previously reported reduction of needle electromyographical paraspinal response related to spinal manipulation delivery. While these large animal in vivo studies investigated various aspects of spinal manipulation dosage on various neurophysiological or biomechanical outcomes, IDP or tissue changes specific to the IVD itself were not investigated. The ability to measure IDP during and after manual therapy procedures in vivo may provide valuable insights into mechanisms associated with subsequent changes in disc hydration/permeability, and/or identifying specific manipulative procedures that maximize/minimize IDP with respect to tolerance/failure of disc tissue in healthy and/or pathological discs. Thus, the purpose of this pilot study was to determine if the feline model was a suitable animal model to record in vivo IDP during spinal manipulation using commercially available spinal manipulation clinical devices (i.e. Activator V and Pulstar) with extremely short thrust durations (2 to 3 ms) [37, 38], as well as during slower spinal mobilization (motorized flexion) movements [39].

## Methods

All animal procedures/protocols were reviewed and approved by the Institutional Animal Care and Use Committee (#20091202 M) with data being collected from three adult male feline preparations (5.3–6.4 kg) that were part of a separate study involving simulated spinal manipulation and recording of trunk muscle spindle response (Oct 2012-May 2013) [40]. General surgical procedures for this preparation have been described in greater detail elsewhere [40–42]. Briefly, anesthesia was induced with isoflurane and maintained with sodium pentobarbital (Nembutal; 35 mg/kg intravenously). Catheters were placed in the common carotid artery and external jugular vein to monitor blood pressure, introduce anesthesia and/or fluids. Animals were mechanically ventilated and arterial gases and body temperature were regularly monitored and maintained within physiological limits (pH 7.32–7.43; PCO2, 32–37 mm Hg; PO2, N85 mm Hg, 38–39 °C). Additional pentobarbital (5 mg/kg) was administered upon withdrawal reflex to noxious pinching of the toe pad or when mean arterial pressure increased above 100 mm Hg.

As part of surgical procedures related to the aforementioned muscle spindle recording study [40], a left paraspinal incision was made exposing the L5 vertebra. An L5 laminectomy was performed exposing the right L6 dorsal rootlets. Paraspinal muscle tissues on the right side remained intact with the exception of a small slit made for attaching toothed forceps to the spinous process to deliver L6 simulated spinal manipulative thrusts using a feedback control motor. All articulating lumbar facet joints and lumbar discs remained intact, however titanium miniscrews (10-mm tomas-pin; Dentaurum, Ispringen, Germany) were placed into left articular pillars at the L4-5, L5-6 and/or L6-L7 levels. After completion of the lumbar muscle spindle data collection, and prior to IDP recording procedures, all lumbar facet screws were removed with facet joints remaining intact. The lumbar intervertebral discs remained unaffected during any of the aforementioned surgical or muscle spindle recording procedures.

**Fig. 1:**
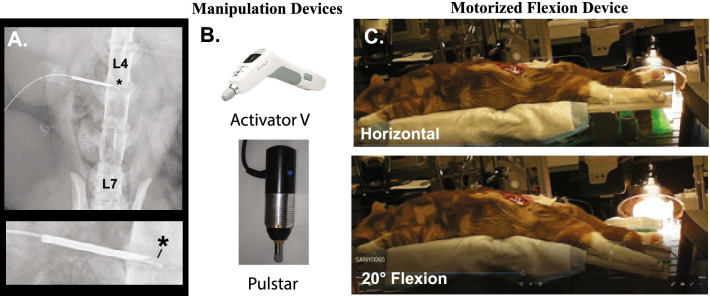
Experimental methodology & manipulation devices. Xray of miniature pressure sensor inserted in the L4-L5 intervertebral disc (**A**) with an enlargement showing the sensor (*) projecting from the tip of a 18 gauge needle (**B**). Photos of commercially available Activator V® and Pulstar® devices that deliver spinal manipulative impulses of 2-3 ms duration. **C**. Photos of anesthetized feline on a hinged motorized flexion device in the horizontal and 20° flexion position

It should be noted that unlike the human spine with five lumbar vertebrae, the feline spine has seven lumbar vertebrae (Fig. 1A). For IVD experiments, the left intervertebral foramen (IVF) was surgically exposed and miniature pressure catheters (Millar SPR-1000) were inserted into either the L4-L5 or L5-L6 disc using an 18 gauge needle (1.26 mm diameter) (Fig. 1A). The dimensions of the needle allowed the miniature pressure catheter to pass through it. The needle shaft had been externally demarcated to indicate desired depth of intradiscal catheter insertion. The needle was slightly withdrawn to expose the pressure sensor (Fig. 1A) while the needle shaft remained in the disc proper (~ 3 mm) for catheter stability purposes. The needle shaft was secured in place with cyanoacrylate. For these pilot experiments, fluoroscopy was not used to visualize needle/catheter placement. Upon completion of the experimental procedures, animals were euthanized by an intravenous overdose of pentobarbital.

### Spinal manipulation devices

Two commercially available hand-held clinical spinal manipulation devices (Activator V® [Activator Methods Int. Ltd., Phoenix AZ], Pulstar® [Sense Technology Inc., Pittsburgh, PA] were used in this pilot study. The Activator V is hand-held clinical device that delivers a solenoid generated mechanical shockwave at 4 progressive force settings (Fig. 1B). The Activator V delivers mean peak forces of 220 N when tested directly on a load cell and 52, 68, 112 and 155 N at settings 1 to 4 when tested against a stiff spinal analog (258.07 N/mm) in handheld operations [43]. Device testing on a more compliant spinal tissue analog (30.22 N/mm) resulted in approximate peak forces of 35, 63, 102 and 130 N at settings 1 to 4 respectively [43]. Prior to Activator V impulse delivery (2 to 3 ms thrust duration), a small preload force (~ 2.9 N) is required and achieved via the rubber tip physically contacting the dorsal surface of the animal. The Pulstar spinal manipulation device (Fig. 1B) has 7 settings and can deliver either single or multiple high velocity mechanical impulses (22 N-155 N when tested directly onto a load cell) after a predefined preload of ~ 15 N has been successfully applied to the device tip [44, 45]. The three lowest force settings on the Pulstar deliver approximate peak forces of 22 N, 44 N, and 67 N respectively with < 5 ms thrust duration. The three lower force settings were the only Pulstar device settings tested in this pilot study. All posterior-to-anterior (90°) manipulative thrusts were delivered directly to the subcutaneous tissue overlying either the L4 or L6 spinous process and the spinal manipulation devices were held by hand during the impulse delivery, simulating clinical application. A series of 3 impulses were delivered with each device with a period of 5 min between a given impulse series at different device settings (equating to 21–28 thrusts delivered per preparation depending on if an additional series of thrusts at sequential device settings was also performed).

### Spinal mobilization device

For spinal mobilization, a custom-made motorized device having a hinged platform in which the caudal portion of the platform moved in computer controlled cyclical oscillatory flexion pattern at 10, 15, 20, 25, or 30 degrees at a fixed rate of 0.14 Hz (Fig. 1C). A series of 5 flexion cycles at each flexion device setting were performed with periods of 5 min between series. This motorized device mimics various clinical spinal flexion or flexion-distraction treatment tables frequently used by chiropractors and/or other manual therapy clinicians in treating low back pain with or without disc involvement. The animal was placed prone on the table platform with the flexion occurring at the level of IDP interest. The vast majority of the animal’s body weight was on the anterior portion of the hinged table requiring no additional stability, while hindlimbs were secured to the rear portion of the hinged table with plastic cable ties to prevent hindlimb slippage off the platform during treatment (Fig. 1C). Similar to that experienced with humans, overall whole-body movement was minimal during flexion-distraction treatment. As often performed clinically during the table flexion phase, a slight cranialward force was manually applied (via thumb contact) to the spinous process of interest to augment distraction of spinal tissues [39]. For this pilot study, these manually applied forces to the spinous process during the flexion phase were not measured.

## Results

**Fig. 2:**
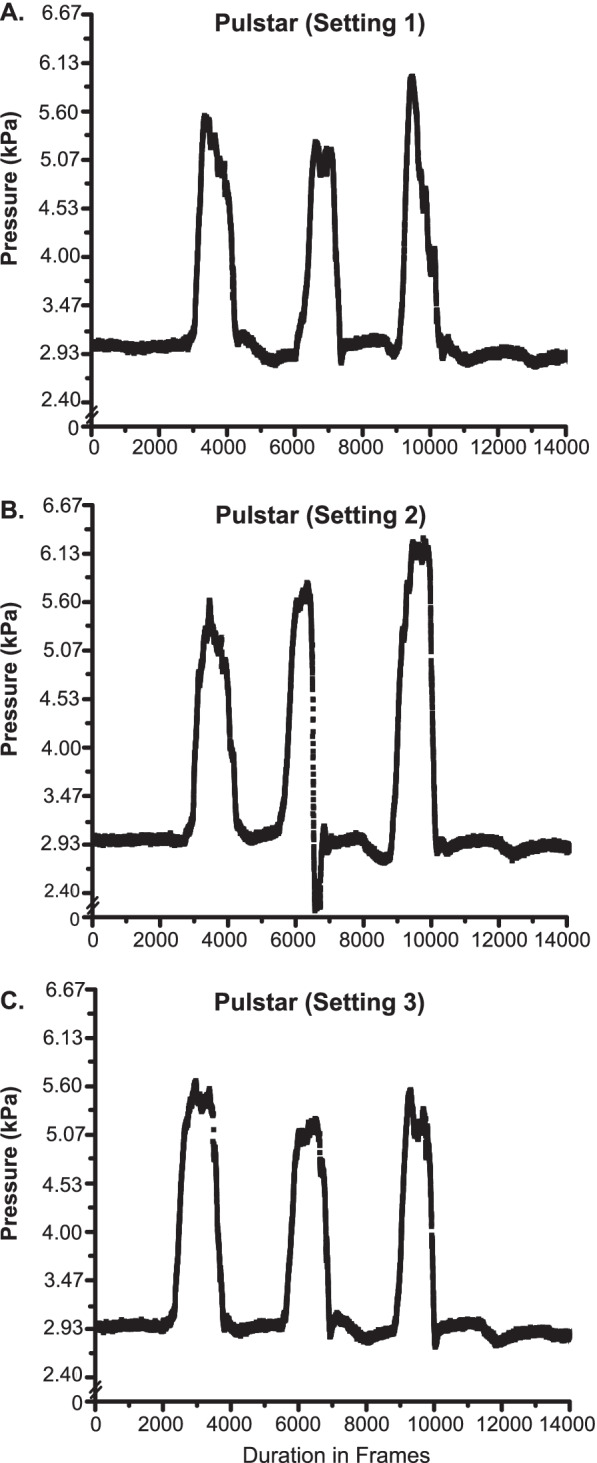
L4-L5 Intradiscal Pressure Changes with the Pulstar Device. Intradiscal pressure (IDP) recordings from the L4-L5 disc during three consecutive Pulstar impulses delivered at device setting 1 (**A**), setting 2 (**B**), and setting 3 (**C**). Note the relatively constant baseline IDP and similar magnitudes of IDP changes across device settings despite a slight increase in peak IDP occurring with the third impulse on settings 1 and 2

Figure 2 shows IDP recordings in a single feline preparation during the delivery of three high velocity low amplitude spinal manipulation impulses delivered to the L4 spinous process using the Pulstar device at settings 1–3. Prone position baseline IDP was ~ 3 kPa for all recordings with an average increase in IDP of ~ 2.5 kPa, ~ 3 kPa, and ~ 2.5 kPa for thrust impulses delivered at settings 1–3 respectively. The three consecutive Pulstar impulses were delivered in fairly rapid succession with the third thrust delivering slightly greater peak IDPs at setting 1 and 2. All three impulses at setting 3 had similar peak IDPs, and IDP changes were equal or slightly less to that of the other two device settings.

**Fig. 3:**
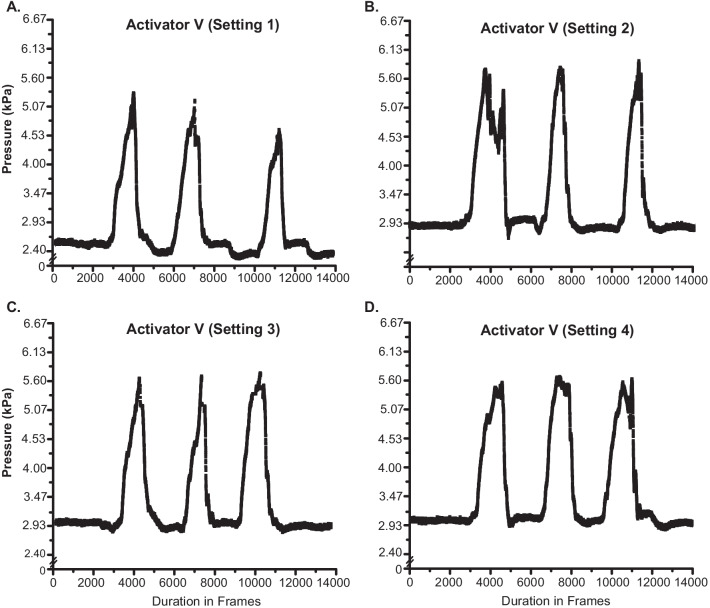
L4-L5 intradiscal pressure changes with the activator V device. Intradiscal pressure (IDP) recordings from the L4-L5 disc during three consecutive Activator V impulses delivered at device setting 1 (**A**), setting 2 (**B**), setting 3 (**C**) and setting 4 (**D**). Note the relatively constant baseline IDP with the exception of setting 1 which was lower and similar magnitudes of IDP changes across device settings. Figures 2, 3 and 4 are from the same experimental preparation

Figure 3 shows IDP recordings during the delivery of three successive manipulation impulses delivered to the L4 spinous process using an Activator V device at settings 1–4 in the same animal as in Fig. 2. Baseline IDP remained at ~ 3 kPa for all recordings with the exception of setting 1 which had a slightly lower IDP baseline of ~ 2.5 kPa. IDP increases of ~ 2.8 kPa, ~ 2.9 kPa, ~ 2.6 kPa, and ~ 2.6 kPa were recorded for Activator V settings 1–4 respectively (Fig. 3). As with the Pulstar device, peak IDP amplitudes remained fairly consistent across the three consecutive thrusts delivered in rapid succession at each of the four Activator V device settings.

**Fig. 4:**
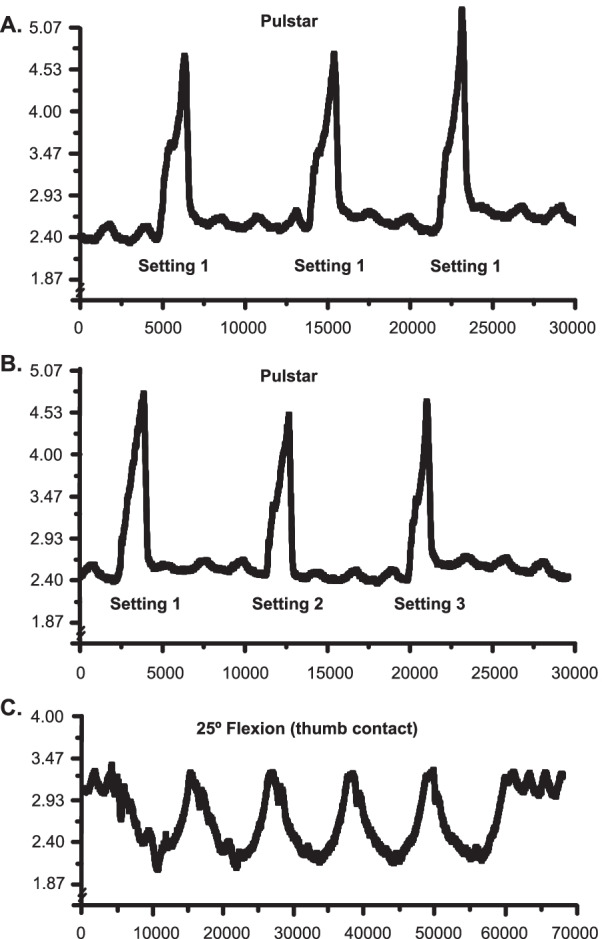
L5-L6 intradiscal pressure changes with pulstar & flexion manipulation devices. Intradiscal pressure (IDP) recordings from the L5-6 disc during three consecutive Pulstar impulses delivered at device setting 1 (**A**), setting 1, 2, 3 (**B**), and 25° motorized flexion with manual thumb contact applying mild cranialward forces to the L6 spinous process (**C**). Note these recordings are from a separate preparation from Fig. 2

Figure 4A and B shows IDP recordings of the L5-6 disc following three L6 spinous process Pulstar impulses in a different animal than earlier recordings presented in Figs. 2 and 3. Prone position baseline IDP was ~ 2.4 kPa and the three Pulstar setting 1 impulses increased IDP changes by ~ 2.4 kPa. These L5-6 IDP changes were similar, but slightly less than that shown in Fig. 2 with thrusts being delivered at the L4 spinous process. As in Fig. 2A, there was also a slight increase in peak IDP during the 3^rd^ thrust. Figure 4B shows the IDP responses following the delivery of L6 three impulses at increasing Pulstar device settings of 1, 2, 3 in this second preparation. IDP changes were relatively consistent (~ 2.4 kPa) across all 3 device settings. Figure 4C shows IDP changes of ~ 1.0 kPa during a series of five motorized 25° trunk flexions with physical contact (thumb) applied cranialward to the L6 spinous process. IDP measurements were consistent across all 5 flexion cycles, with only small increases above baseline IDP during the table’s return to a horizontal position.

**Fig. 5:**
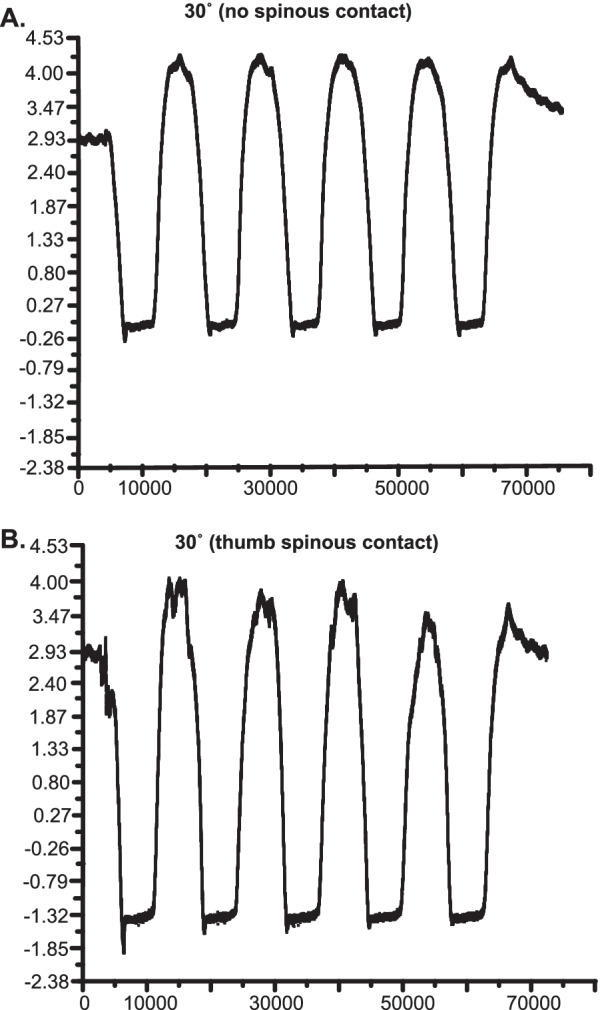
IDP during 30° of motorized flexion with and without manual contact. Intradiscal pressure (IDP) recordings from the L4-L5 disc during motorized flexion of 30° without manual contact (**A**) and with manual thumb contact applied cranialward to the L4 spinous (**B**). Note the additional ~ 1.4 kPa decrease in IDP with applied physical contact

Figure 5 shows a representative example of L4/L5 IDP changes obtained during a series of 5 slow (0.14 Hz) oscillatory trunk flexion movements (30 degrees) without any physical contact on the spinous process (Fig. 5A), as well as with thumb contact to the L4 spinous process (applying slight cranialward forces to the spinous during the table flexion phase; Fig. 5B). Prone position baseline IDP was ~ 2.9 kPa which subsequently decreased by ~ 2.9 kPa without any applied physical contact (Fig. 5A). IDP decreased ~ 4.3 kPa when cranialward forces were manually applied to the L4 spinous process (Fig. 5B). Flexion-related IDP changes were very reproducible regardless of physical contact or the degree of table flexion (10°-30°). L4-L5 IDP transiently increased above baseline values due to the hinged portion of the table going into slight extension (< 5°) upon return to horizontal with and without physical contact (Fig. 5A-B).

## Discussion

To our knowledge this study is the first to report IDP changes related to instrument-assisted spinal manipulation (Activator V, Pulstar) and spinal mobilization procedures in an in vivo animal model. The results of this pilot study demonstrated that an adult feline model is suitable to investigate in vivo IDP changes related to spinal manual therapy in healthy and/or possibly degenerative lumbar disc models. While IDP data was collected from a small number of preparations, they provided insights and generated interesting research questions that require further investigation in relation to the effects that spinal manual therapy has on healthy and degenerative intervertebral discs.

First we noted that for the most part, impulses delivered by the Pulstar and Activator V instruments yielded similar IDP changes (approximately 2.5 to 3.0 kPa; Figs. 2, 3 and 4) despite differences in device design and applied force generation. Mild fluctuations in peak IDP arising during the delivery of three consecutive impulses at a given device setting are most likely the result of the device being handheld creating slight variations in total applied preload forces, as minimum preload forces are required to trigger impulse delivery. In several of the examples shown, we see slight increases in peak IDP associated with the third impulse delivered (Figs. 2A and B, 3B and C), again most likely attributable to the devices being handheld. Despite 3 impulses being delivered in relatively close succession, baseline IDP remained relatively consistent throughout the experimental duration. This indicates very brief, rather than sustained IDP changes occurring in the IVD with instrument-assisted spinal manipulation thrust durations of 2–3 ms. Due to the incompressibility of water, the mechanical shockwaves generated by the thrusts may result in brief pressure spikes related to movement of the water content within the IVD due to hydraulic effects. Baseline IDP was always recorded in the prone position, and typically equated to ~ 5.4% of that recorded in a human. [27] If the average adult cat bodyweight is approximately 3.97 kg [41], this would equate to ~ 4.4% of the average bodyweight of the American male [46] if bodyweight is to be used as a potential scaling tool. However, use of bodyweight as a potential scaling tool can be a rather poor choice as localized thrust forces are quickly dampened by viscoelastic properties of surrounding tissues in vivo and biological cell response does not scale with body weight per se [43, 47]. Compared to testing applied forces directly onto a load cell, spinal manipulation device impulses delivered onto spinal tissue analogs yielded substantially lower (≤ 59%) forces at various device settings [43]. This vast degree of tissue dampening of impulse device applied forces is observed in cadaveric studies, [23, 25] as well in our ongoing in vivo feline studies. Of the 300 N applied to L3/L4 facet joint in a fresh porcine cadaver model, a median peak resultant force of 36.4 N (12.1%) reached the spinal segment [25]. Of these forces reaching the actual spinal segment, the supraspinous ligaments experienced 0.3% of applied peak forces, the facet joint and ligamentum flavum experienced 0.7% of applied peak forces, and the intervertebral disc experienced 99% of peak forces reaching deep tissues [25]. These cadaveric findings highlight the need to determine the degree of viscoelastic tissue diminution of spinal manipulation forces imparted under experimental in vivo conditions. Possibly, a force-feedback controlled device instead of pre-determined settings may allow more control of the applied peak forces and thrust curves. Understanding more with regards to the magnitude of spinal manipulation forces reaching deep tissue structures such as the IVD will help to provide a much more accurate picture of the physiological/biomechanical impact of these clinically applied spinal forces. As previously noted in cadaveric studies [21, 22, 25, 32] and demonstrated in the current in vivo IDP study, specific methods of spinal manual therapy application create unique physiological characteristics within deep spinal structures including the IVD.

Another noteworthy finding from this pilot study that requires greater investigation is that regardless of device used, it appears that minimal IDP changes occur despite the use of higher device settings when compared to the baseline values at the lowest setting (Figs. 2, 3 and 4B). This is interesting and differs from spinal manipulation device testing directly onto load cells which indicate increases in mechanical forces delivered at higher device settings (albeit these increases may not be linear) [48]. This could be related to the inertial effects of the incompressible water contained within the IVD and providing a hydraulic strengthening effect rather than a fluid shift effect. Once a plateau of the hydraulic effects is reached, any additional increase in magnitude of a shockwave would not increase the response. Whether the viscoelastic properties of spinal tissues essentially dissipate any associated increases in applied forces at higher device settings such that IDP remains relatively unchanged will require further investigation, but this initial observation brings up interesting translational questions. Indeed, it may come to be determined that slower application of applied spinal forces are required to achieve IDP changes above a certain physiological threshold.

High-velocity low-amplitude spinal manipulation causes relatively small movements between the manipulated and surrounding vertebrae (between 0.4 and 2.6 mm translation and 0.4–3.5° rotation). While there is greater inherent flexibility in the cat spine compared to the human, spinal stiffness in the intact cat lumbar spine (6.07 to 12.14 N/mm) was similar to that reported following an L5 laminectomy (11.51 N/mm) as tested in the current study. These spinal stiffness measures in the cat lumbar spine are similar to those reported in the rat lumbar spine (14.52 N/mm) and the lumbar spine of healthy human volunteers (~ 11 to 17 N/mm and 14.05 to 16.41 N/mm). That said, larger animal models such as bovine, ovine, or swine should be experimentally considered whenever possible for in vivo IDP research involving manual therapy, as these larger animals will permit manual therapy forces to be applied in similar fashion as that applied in clinical settings and larger animals better mirror human spines biomechanically.

In addition, we also noted the high level of consistency among IDP changes during repetitive spinal mobilization oscillatory cycles with and without physical contact being applied cranialward to the spinous process (Figs. 4C and 5). Delivery of manually-applied forces to the L4 spinous process increased L4-L5 IDP changes by ~ 1.4 kPa in a very consistent and replicable fashion (Fig. 5B). There was an approximate two-thirds reduction in IDP between the L4-L5 and L5-L6 spinal mobilization examples (Figs. 4C, 5). This segmental difference in IDP change could be potentially attributable to several factors including slight proximity differences of the intradiscal pressure sensor to the hinged separation of the flexion table (see Fig. 1C), different segmental anatomy (i.e. thickness of connective tissues), anatomical placements of the IDP sensor into the respective lumbar intervertebral discs, and/or possible sensor cable rotation during catheter placement. However, it should be noted that the Pulstar delivered impulses at both L4 and L6 yielded somewhat similar net IDP changes (Figs. 2 and 4A) suggesting that slight differences in proximity of the pressure sensor to the table center hinge point may have been a larger contributor to segmental IDP differences seen between L4-L5 and L5-6 spinal mobilization examples (Figs. 4C and 5).

## Limitations

There were several limitations in need of acknowledgement in this pilot study. Due to experimental limitations (i.e. animal transport restrictions, experimental barriers, equipment availability/access, etc.), fluoroscopy was not used to guide pressure catheter placement, instead an external marking on the needle shaft served this purpose with direct surgical exposure of the IVF. In future studies, addition of fluoroscopy should be considered as it would better ensure proper catheter placement throughout the duration of the experiment. To help ensure placement stability of the pressure sensor during spinal manipulation procedures, a portion (~ 3 mm) of the 18-gauged needle remained in the disc (Fig. 1A). The disc puncture injury and presence of needle shaft in a portion of the disc had an undetermined effect on disc mechanics during spinal manipulation procedures. Needle puncture injury has been shown to adversely impact disc mechanics in certain studies, [49] while not impacting them in others [50]. It should be noted that needles of similar diameters have been used to insert pressure catheters both in human in vivo studies [28, 29] and animal in vitro studies [51]. It should also be recognized that large differences in disc shape, size, and material composition exists between species, [52–54] therefore caution should always be exercised regarding the generalizability of the results from a feline model to humans and/or other species. Other limitations include that the rate of table flexion was fixed at 0.14 Hz, the impact of different flexion rates on the IDP response was not investigated, rotary and/or other non-posterior-anterior thrust vectors that are often used in clinical settings were not part of the current study, and that forces applied manually to the spinous processes during spinal flexion procedures were not measured in this pilot study. Finally, repeated force application may potentially have affected the mechanical behavior of spinal-related in vivo tissues, so caution should be exercised when interpreting the present findings. Future studies should attempt to address these identified limitations.

## Conclusions

This study marks the first time that in vivo changes in IDP have been reported using clinically available instrument-assisted spinal manipulation devices and/or spinal mobilization procedures. The results of this study indicate that the feline model can be used to investigate IDP changes related to spinal manual therapies, in addition to the diminution of these applied spinal manual therapy forces related to viscoelastic properties of the surrounding spinal tissues. Better understanding of the short and long-term physiological effects of manual therapy on IDP, disc hydration, disc degeneration, and overall disc health may help to inform clinical best practices among manual therapy procedures. Key observations of this pilot study include the high level of IDP reproducibility of spinal manipulation and spinal mobilization procedures with/without manual contact, and the apparent lack of increased IDP changes associated with higher manipulation device settings. These findings alone emphasize the need and potential clinical value of investigating the in vivo relationships between applied spinal manipulative forces and changes in IDP in relation to viscoelastic properties of healthy as well as pathophysiological disc conditions.

## Data Availability

The datasets generated during and/or analysed during the current study are not publicly available due for institutional security reasons but are available from the corresponding author on reasonable request.

## Abbreviations

IDP: Intradiscal pressure
IVD: Intervertebral disc
IVF: Intervertebral foramen

## References

[1] Balague F, Mannion AF, Pellise F, Cedraschi C (2012). Non-specific low back pain. Lancet.

[2] Global, regional, and national incidence, prevalence, and years lived with disability for 354 diseases and injuries for 195 countries and territories, 1990–2017: a systematic analysis for the Global Burden of Disease Study 2017. *Lancet* 2018, **392**(10159):1789–1858. 10.1016/S0140-6736(18)32279-7 PMC6227754 30496104

[3] Cheung KM, Karppinen J, Chan D, Ho DW, Song YQ, Sham P, Cheah KS, Leong JC, Luk KD (2009). Prevalence and pattern of lumbar magnetic resonance imaging changes in a population study of one thousand forty-three individuals. Spine (Phila Pa 1976).

[4] Sizer PS, Phelps V, Matthijs O (2001). Pain generators of the lumbar spine. Pain Pract.

[5] Yamamoto J, Maeno K, Takada T, Kakutani K, Yurube T, Zhang Z, Hirata H, Kurakawa T, Sakai D, Mochida J (2013). Fas ligand plays an important role for the production of pro-inflammatory cytokines in intervertebral disc nucleus pulposus cells. J Orthop Res.

[6] Ma J, Stefanoska D, Grad S, Alini M, Peroglio M (2020). Direct and intervertebral disc-mediated sensitization of dorsal root ganglion neurons by hypoxia and low pH. Neurospine.

[7] Lisi AJ, Holmes EJ, Ammendolia C (2005). High-velocity low-amplitude spinal manipulation for symptomatic lumbar disk disease: a systematic review of the literature. J Manip Physiol Ther.

[8] Coulter ID, Crawford C, Hurwitz EL, Vernon H, Khorsan R, Suttorp Booth M, Herman PM (2018). Manipulation and mobilization for treating chronic low back pain: a systematic review and meta-analysis. Spine J.

[9] Chou R, Deyo R, Friedly J, Skelly A, Hashimoto R, Weimer M, Fu R, Dana T, Kraegel P, Griffin J (2017). Nonpharmacologic therapies for low back pain: a systematic review for an American college of physicians clinical practice guideline. Ann Intern Med.

[10] Pillastrini P, Gardenghi I, Bonetti F, Capra F, Guccione A, Mugnai R, Violante FS (2012). An updated overview of clinical guidelines for chronic low back pain management in primary care. Joint Bone Spine.

[11] Dagenais S, Tricco AC, Haldeman S (2010). Synthesis of recommendations for the assessment and management of low back pain from recent clinical practice guidelines. Spine J.

[12] Wong JJ, Cote P, Sutton DA, Randhawa K, Yu H, Varatharajan S, Goldgrub R, Nordin M, Gross DP, Shearer HM (2017). Clinical practice guidelines for the noninvasive management of low back pain: a systematic review by the ontario protocol for traffic injury management (OPTIMa) collaboration. Eur J Pain.

[13] Kulig K, Powers CM, Landel RF, Chen H, Fredericson M, Guillet M, Butts K (2007). Segmental lumbar mobility in individuals with low back pain: in vivo assessment during manual and self-imposed motion using dynamic MRI. BMC Musculoskelet Disord.

[14] Kulig K, Landel R, Powers CM (2004). Assessment of lumbar spine kinematics using dynamic MRI: a proposed mechanism of sagittal plane motion induced by manual posterior-to-anterior mobilization. J Orthop Sports Phys Ther.

[15] Powers CM, Kulig K, Harrison J, Bergman G (2003). Segmental mobility of the lumbar spine during a posterior to anterior mobilization: assessment using dynamic MRI. Clin Biomech.

[16] Cramer GD, Cambron J, Cantu JA, Dexheimer JM, Pocius JD, Gregerson D, Fergus M, McKinnis R, Grieve TJ (2013). Magnetic resonance imaging zygapophyseal joint space changes (gapping) in low back pain patients following spinal manipulation and side-posture positioning: a randomized controlled mechanisms trial with blinding. J Manipulative Physiol Ther.

[17] Kawchuk GN, Carrasco A, Beecher G, Goertzen D, Prasad N (2010). Identification of spinal tissues loaded by manual therapy: a robot-based serial dissection technique applied in porcine motion segments. Spine (Phila Pa 1976).

[18] Gudavalli MR, Potluri T, Carandang G, Havey RM, Voronov LI, Cox JM, Rowell RM, Kruse RA, Joachim GC, Patwardhan AG (2013). Intradiscal pressure changes during manual cervical distraction: a cadaveric study. Evid Based Complement Alternat Med.

[19] Wu LP, Huang YQ, Zhou WH, Manas D, Zhao WD, Chen JZ, Yin QS, Wang LH (2012). Influence of cervical spine position, turning time, and cervical segment on cadaver intradiscal pressure during cervical spinal manipulative therapy. J Manipulative Physiol Ther.

[20] Gay RE, Ilharreborde B, Zhao KD, Berglund LJ, Bronfort G, An KN (2008). Stress in lumbar intervertebral discs during distraction: a cadaveric study. Spine J.

[21] Funabashi M, Nougarou F, Descarreaux M, Prasad N, Kawchuk GN (2017). Spinal tissue loading created by different methods of spinal manipulative therapy application. Spine (Phila Pa 1976).

[22] Funabashi M, Nougarou F, Descarreaux M, Prasad N, Kawchuk GN (2018). Does the application site of spinal manipulative therapy alter spinal tissues loading?. Spine J.

[23] Funabashi M, Kawchuk GN, Vette AH, Goldsmith P, Prasad N (2016). Tissue loading created during spinal manipulation in comparison to loading created by passive spinal movements. Sci Rep.

[24] Gál J, Herzog W, Kawchuk G, Conway PJ, Zhang YT (1997). Movements of vertebrae during manipulative thrusts to unembalmed human cadavers. J Manipulative Physiol Ther.

[25] Funabashi M, Breen AC, De Carvalho D, Pagé I, Nougarou F, Descarreaux M, Kawchuk GN (2021). Force distribution within spinal tissues during posterior to anterior spinal manipulative therapy: a secondary analysis. Front Integr Neurosci.

[26] Mitchell UH, Helgeson K, Mintken P (2017). Physiological effects of physical therapy interventions on lumbar intervertebral discs: a systematic review. Physiother Theory Pract.

[27] Lisi AJ, O'Neill CW, Lindsey DP, Cooperstein R, Cooperstein E, Zucherman JF (2006). Measurement of in vivo lumbar intervertebral disc pressure during spinal manipulation: a feasibility study. J Appl Biomech.

[28] Sato K, Kikuchi S, Yonezawa T (1999). In vivo intradiscal pressure measurement in healthy individuals and in patients with ongoing back problems. Spine (Phila Pa 1976).

[29] Wilke HJ, Neef P, Caimi M, Hoogland T, Claes LE (1999). New in vivo measurements of pressures in the intervertebral disc in daily life. Spine (Phila Pa 1976).

[30] Wang F, Zhang J, Feng W, Liu Q, Yang X, Zhang H, Han L, Min Y, Zhao P (2018). Comparison of human lumbar disc pressure characteristics during simulated spinal manipulation vs spinal mobilization. Mol Med Rep.

[31] Pickar JG (2002). Neurophysiological effects of spinal manipulation. Spine J.

[32] Funabashi M, Nougarou F, Descarreaux M, Prasad N, Kawchuk G (2017). Influence of spinal manipulative therapy force magnitude and application site on spinal tissue loading: a biomechanical robotic serial dissection study in porcine motion segments. J Manipulative Physiol Ther.

[33] Tawackoli W, Marco R, Liebschner MA (2004). The effect of compressive axial preload on the flexibility of the thoracolumbar spine. Spine (Phila Pa 1976).

[34] Shi C, Qiu S, Riester SM, Das V, Zhu B, Wallace AA, van Wijnen AJ, Mwale F, Iatridis JC, Sakai D (2018). Animal models for studying the etiology and treatment of low back pain. J Orthop Res.

[35] Colloca CJ, Keller TS, Moore RJ, Gunzburg R, Harrison DE (2008). Effects of disc degeneration on neurophysiological responses during dorsoventral mechanical excitation of the ovine lumbar spine. J Electromyogr Kinesiol.

[36] Colloca CJ, Gunzburg R, Freeman BJ, Szpalski M, Afifi M, Moore RJ (2012). Biomechancial quantification of pathologic manipulable spinal lesions: an in vivo ovine model of spondylolysis and intervertebral disc degeneration. J Manipulative Physiol Ther.

[37] Reed WR, Liebschner MA, Sozio RS, Pickar JG, Gudavalli MR (2015). Neural response during a mechanically assisted spinal manipulation in an animal model: a pilot study. J Nov Physiother Phys Rehabil.

[38] Reed WR, Pickar JG, Sozio RS, Liebschner MAK, Little JW, Gudavalli MR (2017). Characteristics of paraspinal muscle spindle response to mechanically assisted spinal manipulation: a preliminary report. J Manipulative Physiol Ther.

[39] Onifer SM, Reed WR, Sozio RS, Long CR (2015). Antinociceptive effects of spinal manipulative therapy on nociceptive behavior of adult Rats during the formalin test. Evid Based Complement Alternat Med.

[40] Reed WR, Pickar JG (2015). Paraspinal muscle spindle response to intervertebral fixation and segmental thrust level during spinal manipulation in an animal model. Spine (Phila Pa 1976).

[41] Reed WR, Cao DY, Long CR, Kawchuk GN, Pickar JG (2013). Relationship between biomechanical characteristics of spinal manipulation and neural responses in an animal model: effect of linear control of thrust displacement versus force, thrust amplitude, thrust duration, and thrust rate. Evid Based Complement Alternat Med.

[42] Pickar JG (1999). An in vivo preparation for investigating neural responses to controlled loading of a lumbar vertebra in the anesthetized cat. J Neurosci Methods.

[43] Liebschner MA, Chun K, Kim N, Ehni B (2014). In vitro biomechanical evaluation of single impulse and repetitive mechanical shockwave devices utilized for spinal manipulative therapy. Ann Biomed Eng.

[44] Leach RA, Parker PL, Veal PS (2003). PulStar differential compliance spinal instrument: a randomized interexaminer and intraexaminer reliability study. J Manipulative Physiol Ther.

[45] Evans J (1998). Differential compliance measured by the function recording and analysis system in assessment of vertebral subluxation. J Vert Sublux Res.

[46] Center for Disease Control and Prevention-National Center for Health Statistics. Body Measurements U.S., https://www.cdc.gov/nchs/fastats/body-measurements.htm (July 29, 2022).

[47] Liebschner MA (2004). Biomechanical considerations of animal models used in tissue engineering of bone. Biomaterials.

[48] Colloca CJ, Keller TS, Black P, Normand MC, Harrison DE, Harrison DD (2005). Comparison of mechanical force of manually assisted chiropractic adjusting instruments. J Manipulative Physiol Ther.

[49] Elliott DM, Yerramalli CS, Beckstein JC, Boxberger JI, Johannessen W, Vresilovic EJ (2008). The effect of relative needle diameter in puncture and sham injection animal models of degeneration. Spine (Phila Pa 1976).

[50] Hwang D, Gabai AS, Yu M, Yew AG, Hsieh AH (2012). Role of load history in intervertebral disc mechanics and intradiscal pressure generation. Biomech Model Mechanobiol.

[51] Bashkuev M, Vergroesen PA, Dreischarf M, Schilling C, van der Veen AJ, Schmidt H, Kingma I (2016). Intradiscal pressure measurements: a challenge or a routine?. J Biomech.

[52] Beckstein JC, Sen S, Schaer TP, Vresilovic EJ, Elliott DM (2008). Comparison of animal discs used in disc research to human lumbar disc: axial compression mechanics and glycosaminoglycan content. Spine (Phila Pa 1976).

[53] Showalter BL, Beckstein JC, Martin JT, Beattie EE, Espinoza Orías AA, Schaer TP, Vresilovic EJ, Elliott DM (2012). Comparison of animal discs used in disc research to human lumbar disc: torsion mechanics and collagen content. Spine (Phila Pa 1976).

[54] O'Connell GD, Vresilovic EJ, Elliott DM (2007). Comparison of animals used in disc research to human lumbar disc geometry. Spine (Phila Pa 1976).

